# MED38/465: Innovative Medical Education in an Integrated Framework of Case-Based Learning and Web-Based Training

**DOI:** 10.2196/jmir.1.suppl1.e74

**Published:** 1999-09-19

**Authors:** R Singer, J Riedel, FJ Leven

**Affiliations:** ^1^Hygiene InstitutLabor Computerunterstützte Ausbildung in der MedizinHeidelberg, HeilbronnGermany

**Keywords:** Java, CBT, WBT, Case Simulation, Internet

## Abstract

**Introduction:**

With increasing network bandwidth and computing power the Internet will become more and more important in education. Web-Based Training (WBT) Systems leverage the advantage of flexible learning with respect to training systems being platform-independent, adaptive, easy to install, and update, easy to administer. Using standards like HTML and Java, the internet offers many resources world-wide, that can be accessed from inside WBT-Systems. As part of the Virtual University approach VIROR, the laboratory for Computer-Based Training in medicine " at the University of Heidelberg is developing the Web-Based Training-System CAMPUS, which integrates medical cases with systematic knowledge from many different sources.

**Methods:**

Main components of CAMPUS are the authoring system, the presentation component, and a repository for systematic medical knowledge. The authoring tool is used by a medical author for editing medical cases. The presentation tool can be used as a case simulator as well as for case retrieval. During case simulation, the student is able to get further information, to specific situations within a case. This means, he has the possibility to jump from every case-situation to integrated encyclopaedias, digital libraries, and databases (like Medline or Cochrane) to get enough knowledge to solve the actual case problem. To build a bridge between medical cases and systematic knowledge, CAMPUS takes advantage of MeSH as a medium for semantic interoperability. With the author assigning MeSH-Codes to particular case-objects, the presentation component is able to jump directly to the specific topic in the systematic knowledge-base or encyclopaedia. The system is based on a 7 tier architecture and is fully implemented in Java as a client-server architecture using Java RMI for communication. The central knowledge base will be implemented as an XML repository, and will be enriched by resources in the internet. Medical cases as well as the simulation logic are stored in a relational database.

**Results:**

At present, prototypes of the CAMPUS authoring-system and presentation component are available and the first microbiology and pediatric cases are implemented in the system. In the future, primary efforts will be made in acquiring new medical cases as well as systematic knowledge.

Discussion;

CAMPUS has a high degree of adaptiveness and presents a patient in a realistic way using the advantages of Web-based Training. It is being developed in close co-operation with instructional psychologists and medical faculty members who want to integrate the system in the curriculum of medicine at the University of Heidelberg.

